# Indoor green wall affects health-associated commensal skin microbiota and enhances immune regulation: a randomized trial among urban office workers

**DOI:** 10.1038/s41598-022-10432-4

**Published:** 2022-04-20

**Authors:** L. Soininen, M. I. Roslund, N. Nurminen, R. Puhakka, O. H. Laitinen, H. Hyöty, A. Sinkkonen, ADELE research group, Damiano Cerrone, Mira Grönroos, Nan Hui, Anna Luukkonen, Iida Mäkelä, Noora Nurminen, Sami Oikarinen, Anirudra Parajuli, Riikka Puhakka, Marja I. Roslund, Mika Saarenpää, Laura Soininen, Yan Sun, Heli K. Vari, Olli H. Laitinen, Juho Rajaniemi, Heikki Hyoty, Aki Sinkkonen

**Affiliations:** 1 Ecosystems and Environment Research Programme, Faculty of Biological and Environmental Sciences, University of Helsinki, Niemenkatu 73, 15140 Lahti, Finland; 2 Faculty of Medicine and Health Technology, Tampere University, Arvo Ylpön katu 34, 33520 Tampere, Finland; 3 Natural Resources Institute Finland, Horticulture Technologies, Turku and Helsinki, Finland

**Keywords:** Microbial ecology, Biodiversity

## Abstract

Urbanization reduces microbiological abundance and diversity, which has been associated with immune mediated diseases. Urban greening may be used as a prophylactic method to restore microbiological diversity in cities and among urbanites. This study evaluated the impact of air-circulating green walls on bacterial abundance and diversity on human skin, and on immune responses determined by blood cytokine measurements. Human subjects working in offices in two Finnish cities (Lahti and Tampere) participated in a two-week intervention, where green walls were installed in the rooms of the experimental group. Control group worked without green walls. Skin and blood samples were collected before (Day0), during (Day14) and two weeks after (Day28) the intervention. The relative abundance of genus *Lactobacillus* and the Shannon diversity of phylum Proteobacteria and class Gammaproteobacteria increased in the experimental group. Proteobacterial diversity was connected to the lower proinflammatory cytokine IL-17A level among participants in Lahti. In addition, the change in TGF-β1 levels was opposite between the experimental and control group. As skin *Lactobacillus* and the diversity of Proteobacteria and Gammaproteobacteria are considered advantageous for skin health, air-circulating green walls may induce beneficial changes in a human microbiome. The immunomodulatory potential of air-circulating green walls deserves further research attention.

## Introduction

Due to an increased hygiene level^1^, biodiversity loss and irregular soil contacts^2–4^ the exposure to environmental microbes has reduced in Western cities, which is seen as one of the major reasons for the rise in immune-mediated diseases, such as autoimmune diseases and allergies^5^. Nature-derived microbes that have a commensal relationship with humans contribute to the development and regulation of the human immune system^1,4,6–8^. The skin microbiome can be altered via skin contact to microbial sources and hands are a common route of microbial transmission^9–13^. Indoors, humans affect and are exposed to microbial communities by touching indoor surfaces^14^. In addition to direct skin contact, humans are exposed to and affected by microbes in the air, for example, via skin and airways^15–17^.

Each city has its own unique microbiome^18,19^ and its composition in the soil^20^ and in the air^21^ is affected by vegetation. Indeed, plant surfaces are a known source of airborne bacteria^17,22,23^. Additionally, indoor plants increase the abundance and diversity in bacterial communities on indoor surfaces^24^. The amount of vegetated area in the locality affects the odds of developing immune-mediated diseases as a child^25–27^. Vegetation also affects the composition of the human gut microbiome, which impacts human immunoregulation^28^. Previous research has identified certain bacterial groups that are abundant in soil and vegetation as indicators of a healthy skin microbiome. For example, Proteobacteria belongs to the most common phyla on human skin (relative abundance > 5%); its diversity and relative abundance seems to have a role in human immune regulation^4,12,25^. The diversity and relative abundance in bacteria belonging to the class Gammaproteobacteria is an indication of health on human skin^4,7^ and on plants^29^. Additionally, bacteria belonging to the genus *Lactobacillus* on skin fend off pathogens and better the integrity of skin^30^; some *Lactobacillus* species found on humans also occur on plants^31,32^.

Due to poor microbiological assemblages in cities, urbanization reduces indoor microbial diversity^33–35^. Microbial assemblages can be affected with urban gardening^36^ and urban greening^12,37,38^. In previous studies, the effects of soil and plant-based biodiversity interventions have been observed in human subjects as bacterial changes in skin and stool microbiome, and altered immunoregulatory cytokine levels in the blood^9,11,12^. Surprisingly, hardly any studies survey whether indoor greening shapes commensal microbiota and immune response among urban dwellers^24^.

The current study explored if bacterial communities in the human subjects spending time indoors can be altered via vegetated walls that circulate indoor air. For the intervention, vegetated walls (green walls) were brought into offices of university personnel for two weeks and the impact was investigated via skin and blood samples. The study subjects were expected to be exposed to the green walls via microbial communities in the air and on indoor surfaces but not by touching the green walls. The microbial focus was on the bacterial alpha diversity and the relative abundance of health-associated proteobacterial taxa and *Lactobacillus* on skin. To observe possible immune responses, the levels of the anti-inflammatory cytokines interleukin 10 (IL-10) and transforming growth factor– β1 (TGF-β1)^39,40^, and the proinflammatory cytokine interleukin 17A (IL-17A)^41^ were measured from the blood samples. Immunomodulatory pathways respond to IL-10 concentration in the blood and IL-10 has been researched for therapeutic use in immunomodulation^9,12^ and prevention of immune-mediated diseases, such as inflammatory bowel disease and rheumatoid arthritis^40^. Similarly, TGF-β1 is connected to several immune-mediated diseases as an inhibitor and has an essential impact on all types of immune cells^42–44^. The upregulation of cytokines in the IL-17 family in turn, seem to advance the pathogenesis of immune-mediated diseases^41,45^. We hypothesized that the intervention would increase the relative abundance and alpha diversity of health-associated taxa on the skin and affect the levels of the measured immune system cytokines.

## Materials and methods

### Green walls

The green walls (size 2 m × 1 m × 0.3 m) used in this study were Naava One (Naava, Jyväskylä, Finland; www.naava.io) that circulate indoor air. They first absorb the indoor air through the plant roots and soilless substrate, then automated fans circulate the air back to the room. When the indoor air passes through the green wall, volatile organic compounds (VOC) are efficiently removed via biofiltration by microbes, plants and the growing medium^46^. The watering system is automated and the water circulates within the wall. Each green wall contains three plant taxa (heartleaf philodendron (*Philodendron scandens*), dragon tree (*Dracaena* sp*.*) and bird’s nest fern (*Asplenium antiquum*) growing altogether in 63 units. Each unit consists of two to four plant individuals.

### Treatment groups and sample collection

The study (a randomized controlled trial with parallel design) was conducted in offices of university personnel in two Finnish cities (Lahti and Tampere). The study followed the recommendations of Finnish Advisory Board on Research Integrity, and it was approved by the ethics committee of the local hospital district (Hospital District of Pirkanmaa, Finland). A written informed consent in accordance with the Declaration of Helsinki was signed by all participants. The study subjects were healthy adults. The exclusion criteria were age below 18 at the beginning of the study, daily smoking, immune deficiency (e.g., antibody deficiency, HIV infection), immunosuppressive medication (e.g., corticosteroids), a condition affecting immune response (e.g., rheumatoid arthritis, colitis ulcerosa, Crohn’s disease, diabetes, and Down syndrome), or cancer diagnosis. All volunteers that filled the inclusion criteria were accepted to the study.

The resulting 28 study subjects were randomly divided (intended allocation ratio 1:1; simple randomization done by an independent researcher at University of Helsinki; mechanism: random number table) into two treatment groups that were the control group (without green wall exposure) and the experimental group exposed to green walls (Table 1). After the randomization, it was ensured that age and sex ratio were similar in both groups, and no changes were needed. The final allocation ratio was 17:11 in the control and the experimental group. The study subjects in the green wall group received a green wall in the office rooms and were exposed to the green walls only at the office during their workdays. The study was implemented in two buildings in Lahti and one building in Tampere, Finland (Table 1). All study subjects answered surveys about their living conditions and history (such as type of housing, pets and land use type in their locality) and their living habits during the experiment on Day14 and Day28 (such as hours spent in nature, travel, medication, illnesses and food supplements). Depending on the office room size, 1–2 green walls were installed in the treatment office rooms in Tampere and Lahti for two weeks, according to instructions of the manufacturer (www.naava.io). When the room size was more than 60 m^2^, two green walls were used as instructed by the manufacturer.

**Table 1:** Characteristics of treatment groups: Ctrl = control, Exp = experimental.

Groups		Ctrl	Exp
	Participants	17	11
Sex	Female	13	9
	Male	4	2
Age	25–35	3	4
	36–45	7	2
	36–45	5	5
	Average	40	40
	SD	9	10
Type of residence	Apartment building	4	4
	Rowhouse	2	2
	Private house	10	5
Work place	Lahti	10	7
	Tampere	4	7

Skin and blood samples were collected from both experimental and control group participants before installing the green walls (Day0), on the last day of the intervention (Day14) and two weeks after the intervention (Day28) by trained nurses as described by Roslund et al.^12^. Briefly, skin samples were collected by swabbing an area of 5 cm–by–5 cm on the back of the palm for 10 s. The swabs were wetted with saline buffer (0.1% Tween 20 in 0.15 M NaCl) before sample collection, and after sampling the cotton tips were cut off into sterile polyethene tubes and stored at − 80 °C until analysis. Venous blood was collected into Vacutainer CPT Mononuclear Cell Preparation tubes containing sodium citrate (BD Biosciences, NJ, USA) and centrifuged according to the manufacturer's instructions to separate the plasma and the plasma samples were stored at − 80 °C until analysis.

### Skin and blood sample processing

The skin samples were prepared for bacterial DNA sequencing as in Roslund et al.^47^. The bacterial DNA was extracted from the skin swabs with Fast DNA spin kit for soil (MP biomedicals, Santa Ana, CA) according to the manufacturer's protocol. The DNA concentration was quantified by Quant-iTTM PicoGreen® dsDNA reagent kit (Thermo Fisher Scientific, Waltham, MA, USA). The DNA concentration in the samples was adjusted to 0.4 ng/ml before polymerase chain reaction (PCR) with which variable region V3-V4 within the 16S ribosomal RNA (rRNA) gene was amplified. Forward primer was 515F 50- GTGCCAGCMGCCGCGGTAA-30 and reverse primer 806R 50- GGACTACHVGGGTWTCTAAT-30 with truncated Illumina overhangs as in Hui et al.^37^. Negative controls for DNA extraction (sterile water) and PCR (no sample) were sequenced with the samples. Positive control for PCR was made using (*Cupriavidus necator* JMP134, DSM 4058). Success of amplification process was confirmed with agarose gel (1.5%) electrophoresis. The primers were cleaned from the PCR products with Agencourt AMPure XP solution (Beckman Coulter Inc., Brea, CA, USA). The samples were sequenced with Illumina MiSeq 16S rRNA gene metabarcoding with read length 2 × 300 base pairs using a V3-V4 reagent kit at the Institute for Molecular Medicine Finland (FIMM, Helsinki, Finland).

The concentration of cytokines IL-17A and IL-10 were measured from the plasma samples using Milliplex MAP high sensitivity T cell panel kit (Merck KGaA, Darmstadt, Germany) with Bio-Plex® 200 system (Bio-Rad Laboratories, Hercules, CA, USA) and Bio-Plex Manager software (version 4.1, Bio-Rad Laboratories, Hercules, CA, USA). TGF-β1 concentration was analyzed using ELISA (BioVendor, Czech Republic).

### Bioinformatics

From the skin samples’ sequence data, the bacterial OTUs were identified to the genus level according to studies by Schloss et al.^48^ and Kozich et al.^49^ as in Soininen et al.^50^. Briefly, using Mothur (version 1.44.1) the sequences were aligned with SILVA (version 138)^51^ as a reference. The sequences were preclustered to avoid sequencing errors^52^. Chimeras were searched by UCHIME^53^ and deleted. The sequences were classified using Bayesian classifier^54^ with SILVA (version 138)^51^ with 80% bootstrap threshold. Non-bacterial sequences were deleted. Unique sequences were clustered to OTUs at 97% sequence similarity. OTUs with 10 sequences or less were removed. Good’s coverage index (average ± SD: 0.98 ± 0.01) and alpha diversity indices were calculated for each sample using summary.single command. These calculations and the subsampling of the samples were done according to the smallest sequence count (3893) in the samples. Contaminant OTUs were removed as in Roslund et al.^13^. Abundant bacterial taxa (relative abundance of > 0.01%) were selected for further analyses. Alpha diversity indices for phylum Proteobacteria, class Gammaproteobacteria and genus *Lactobacillus* were calculated from the subsampled data using R version 3.6.1^55^ function *diversity* of package *vegan*^56^.

### Outcome measures and sample size estimation

Primary outcome measure was Alpha diversity of skin Gammaproteobacteria, since it was associated with environmental biodiversity, and TGF-β in a previous study^12^. Gammaproteobacteria Shannon diversity index was measured at baseline and after 28-day intervention. Secondary outcome measure was relative abundance of skin Lactobacillus and cytokine levels measured from plasma. All the secondary outcome measures were analyzed from baseline to end of intervention. No side effects were observed.

The primary outcome measure for the power calculation was the difference between intervention and control study subjects in the change of Gammaproteobacterial diversity on the skin between baseline and day 28. We used prior effect estimates from the study that estimates correlations between environmental biodiversity, human microbiota and immune function^12^. In this study, the alpha diversity of Gammaproteobacteria was higher among study subjects in the intervention arm in more biodiverse environment, and Gammaproteabacterial abundance on skin was associated with TGF-β expression. Generic diversity of Gammaproteobacteria among study subjects in contact with green materials (intervention arm) had an average of 17 Gammaproteobacterial genera in their hands and a standard deviation of 5, whereas in the hands of study subjects in the urban control arm they had an average of 8 and a standard deviation of 5. When the significance level is set to *P* ≤ 0.05 and the statistical force is 0.8 (80.1%), the between-cluster (between cities) coefficient of variation is 0.2, the required sample size for each group is 14.

### Statistics

Bacterial diversity and relative abundance (dependent variables) of selected taxa were tested statistically in contrast to timepoint and treatment (explanatory variables) using linear mixed models (LMMs) (function lmer in lme4 package in R) with study subject (nested in cities) as the random factor. LMMs are a good fit for analyzing clustered data and by using study subject as the random factor, the fact that one person is the source for several samples, can be taken into account in the statistical evaluation^57,58^. Additionally, the amount of change (between timepoints Day0–Day14 and Day0–Day28)^59^ in diversity and relative abundance were calculated and compared using LMMs as in Roslund et al.^12^. Additionally, the treatments were compared on each timepoint separately using t-test or Wilcoxon test depending on the Shapiro–Wilk distribution of the variable. The cytokine levels and their changes (dependent variables) were tested in contrast to bacterial values, the interaction of timepoint and treatment (explanatory variable) using LMMs with study subject (nested in cities) as the random factor. The background information and living habits were compared between the treatments using Chi-Square test for nominal data and t-test or Wilcoxon’s test for quantitative data.

## Results

**Figure 1:**
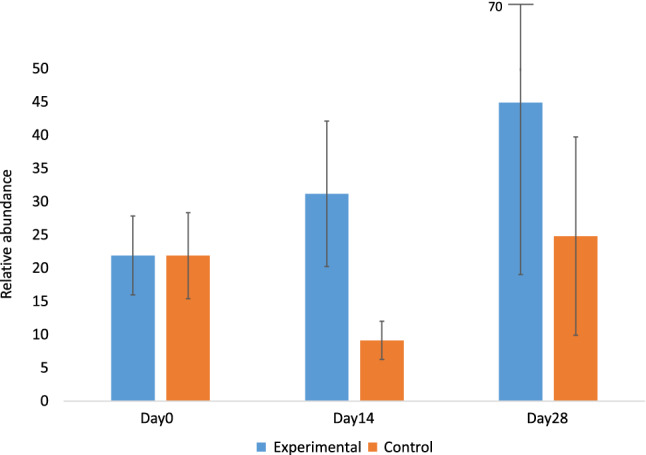
Relative abundance of Lactobacillus spp. (mean ± SE). The relative abundance was calculated by subsampling to the lowest sequence count in the samples (3893). The relative abundance on Day14 (Wilcoxon *P* = 0.0058) and the amount of change (Day14–Day0) was higher in the experimental group.

The relative abundance of *Lactobacillus* spp. (Fig. 1 and Supplementary Table 1) was higher in the skin samples of the experimental group than the control group during the treatments, on Day14 (Wilcoxon *P* = 0.0058). Additionally, the change (Day14 – Day0) in the relative abundance of *Lactobacillus* spp. was higher in the experimental group than in the control group. Within the experimental group, the relative abundance of *Lactobacillus* spp. increased in six study subjects and decreased in three study subjects. Within the control group, the relative abundance decreased in 13 study subjects and increased in three study subjects. Importantly, random variation between individuals explains total variation only partially (LMM All: *P* < 0.001, R2 = 0.05, R2 random = 0.21). The significance of the model did not depend on the city (LMM Lahti *P* < 0.001, R2 = 0.05, R2random = 0.31; LMM Tampere *P* < 0.001, R2 = 0.25, R2random = 0.06).

**Figure 2:**
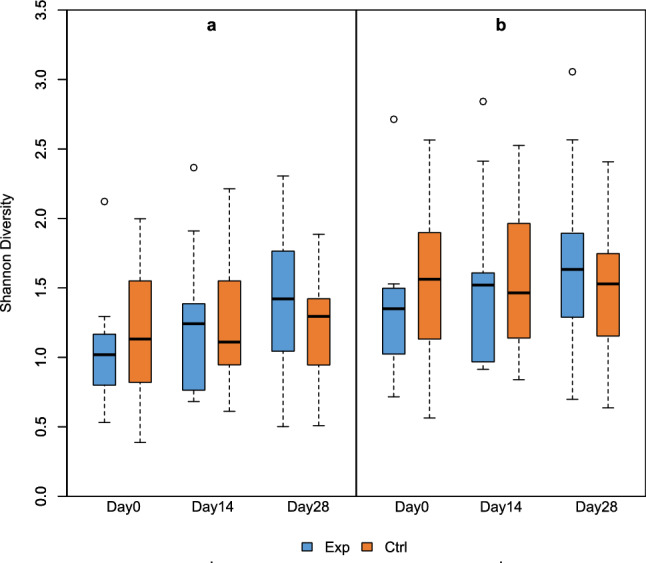
Shannon diversity index of class Gammaroteobacteria (**a**) and phylum Proteobacteria (**b**) on days 0, 14 and 28 for the experimental group (Exp) and the control group (Ctrl). The change in Shannon diversity (Day28–Day0) differed between treatment groups in Proteobacteria and (LMM: *P* = 0.04, R2 = 0.02, R2random = 0.67) and Gammaproteobacteria (LMM: *P* = 0.02, R2 = 0.03, R2random = 0.66).

There were subtle differences in the Shannon diversity of Gammaproteobacteria (Fig. 2A and Supplementary Table 1) and Proteobacteria (Fig. 2B and Supplementary Table 1). The change in Shannon diversity (Day28 – Day0) differed between treatment groups in Proteobacteria, plausibly due to high Day28 values in the experimental group (LMM: *P* = 0.04, R2 = 0.02, R2random = 0.67) and Gammaproteobacteria (LMM: *P* = 0.02, R2 = 0.03, R2random = 0.66). Interestingly, the diversity changes in Proteobacteria were dominant among participants in Lahti but not in Tampere (Supplementary Fig. 1).

**Figure 3:**
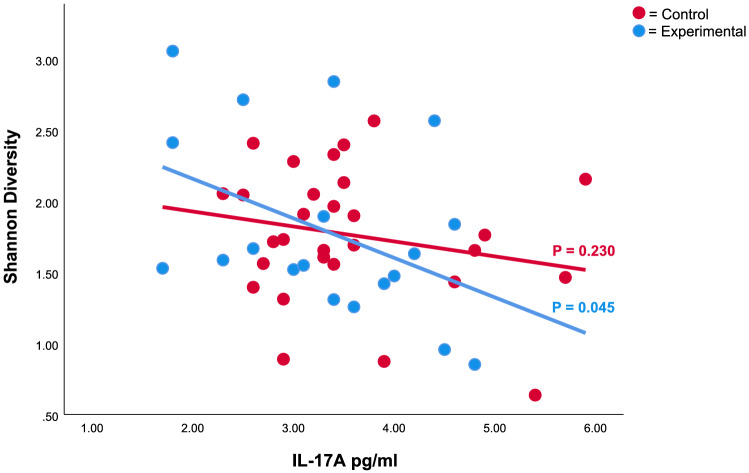
Shannon diversity (y-axis) in phylum Proteobacteria on skin associated with IL-17A concentration (pg/ml) in blood (x-axis) among Lahti participants. High Shannon diversity of Proteobacteria was associated to a low levels of proinflammatory IL-17A concentration (LMM: P = 0.017, R2 = 0.10, R2random = 0.029).

Among Lahti dwellers, low cytokine IL-17A levels were associated to high Shannon diversity in class Gammaproteobacteria and phylum Proteobacteria (Fig. 3 and Supplementary Table 2) with time as the random factor. The association with Gammaproteobacteria was observed when both treatment groups were included in the model (LMM: *P* = 0.04, R2 = 0.08, R2random = 0.02). The association with Proteobacteria was observed when both treatment groups were included (LMM: *P* = 0.017, R2 = 0.10, R2random = 0.029; Fig. 3) and within the experimental group (LMM: *P* = 0.045, R2 = 0.19, R2random = 0).

**Figure 4:**
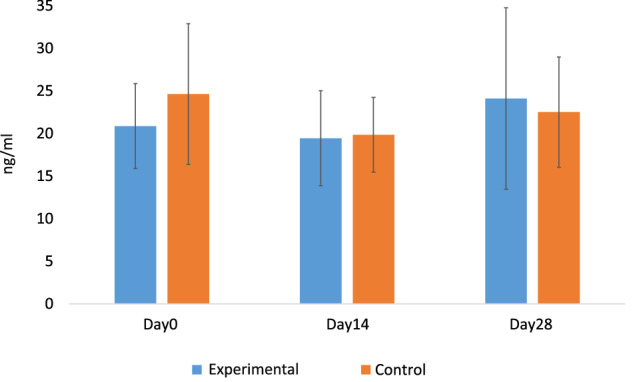
The concentration of the cytokine TGF-β1 (ng/ml) increased in the experimental group and decreased in the control group on Day28 in comparison to Day0 (LMM: P = 0.04, R2 = 0.08, R2random = 0.52) in the level of change in the anti-inflammatory cytokine TGF-β1 on Day28 (Day28–Day0).

The study groups differed significantly (LMM: *P* = 0.04, R2 = 0.08, R2random = 0.52) in the level of change in the anti-inflammatory cytokine TGF-β1 on Day28 (Day28 – Day0). The concentration of TGF-β1 (Fig. 4 and Supplementary Table 1) increased in the experimental group and lowered in the control group. According to the R2 –values, location (city) explains the result more (52%) than the treatment (8%) (Supplementary Fig. 2). In IL-10 levels, there were no significant changes in connection to the treatments or health-associated bacterial taxa. Regarding the living habits during the experiment and the background information, there were no significant differences found between the treatment groups.

## Discussion

The changes observed in this green wall study were connected to Proteobacteria and *Lactobacillus* that have been shown to be beneficial for human health. As far as we are aware of, this is the first study that shows a change in the relative abundance of *Lactobacillus* spp. on skin in response to green wall exposure. The bacteria from *Lactobacillaceae* family (such as *Lactobacillus* spp.) are known to act against pathogens and inflammation on skin^30,60^. Their application as a probiotic on skin has been recommended in the treatment of sunburns^61^, skin oxidative damage and hyperpigmentation^62^. Therefore, the observed steady and continuous increase in the relative abundance of skin *Lactobacillus* is an important finding. Spending time in green wall rooms seems to be related to increasing abundance of health-supporting skin microbiota within a relatively short time period of two weeks. This support health benefits of working in rooms having green walls with air circulation system; usually green wall are long-term interior design elements.

The diversity of Proteobacteria and Gammaproteobacteria has been observed to be higher among healthy people compared to people with immune-mediated diseases such as atopy and allergies^4,7,25^. The diversity of Gammaproteobacteria on skin has successfully been altered via biodiversity intervention with an impact to immune regulation^12,13^. The elevation in the diversity of proteobacterial taxa on the skin of participants working in the green wall offices of this study makes sense because Proteobacteria are a common part of plant microbiomes. However, the elevation was observed only in Lahti (17 study subjects), and according to the R2 values regarding proteobacterial taxa, city as a factor had a high effect on the results. As seen in Fig. 3, the plausible reason for the difference in IL-17A level is the increasing proteobacterial abundance in Lahti experimental group. An interesting detail is that graphically even Day0 values were slightly higher in Lahti, though there were no statistical difference (Fig. 3). In Tampere, all study rooms were situated in the area of Tampere University Hospital, whereas in Lahti the study rooms were at two separate campus areas (in the city center and between industrial areas) without a connection to medical sciences. Therefore, the daily hygiene practices were probably different between the office workers at the medical campus in Tampere and the two non-medical campuses in Lahti. Due to the differences in the location, the surroundings of the study buildings may also have different hygiene levels which affects microbial diversity^1,4^. Further, the building in Tampere was built in 2016 whereas the buildings Lahti were considerably older (built 1993 and 1980); the age of a building affects the indoor microbiome composition^26^. A third, potentially parallel explanation is that microbial communities in offices are city-specific^18,19^; it is tempting to speculate whether the impact of city is strong enough to mask subtle changes in the relative abundance of Proteobacteria.

The current study was not designed to explore the mechanisms that lead to changes in skin microbiota. Our hypothesis is that green walls balance air moisture and release spores or live bacteria that land on skin^17^. However, we cannot separate the role of the introduced microbiome from the green walls from the consequences of the removal of volatile organic compounds (VOC) by the green walls; the green walls used in this study remove VOCs^46^. Since VOCs are known to affect the composition and processes of bacterial communities in the environment^63,64^ and on skin^65^, the green walls could have an indirect impact to indoor and skin bacterial communities. VOCs include pollutants released from materials used in interior decoration^46^ but they also include compounds emitted by organisms which may use them for interaction^65^. For example, skin bacteria may inhibit one another via VOCs^66^. Therefore, the green walls may remove VOCs that would otherwise impact the bacterial communities indoors and on skin. To distinguish the mechanism responsible for altered skin microbiota, the microbiome of the green walls should be sampled and the VOC composition in the study rooms should be analyzed.

Since IL-17A is a proinflammatory cytokine associated with adverse health outcomes, like low-grade inflammation^67^, the association between the high proteobacterial diversity and the low IL-17A concentration seems beneficial. In addition, the change of anti-inflammatory cytokine TGF-β1 in the experimental group on Day28 seems beneficial due to the gain of concentration. As with bacterial results, the cytokine results were impacted by the random factors (city and study subject). Individual differences are typically large when the study subjects live outside lab conditions. However, this does not diminish the importance of the observed difference between the experimental and control group; based on our results, air-circulating green walls change skin microbial communities among urban dwellers.

Although access to nature outside workhours was permitted, the hours spent in nature was surveyed on Day14 and Day28 and no difference was found between the experimental and control group. Therefore, it seems unlikely that free time in nature was sufficient to overcome the effect of green walls. The access to other study offices was not restricted (contamination) but visits to other offices were either very short or nonexistent; the typical places of interaction were the coffee rooms.

Based on our findings, air-circulating green walls alter the microbiome and modulate the immune system among office workers. Air-circulating green walls have potential in promoting microbiological diversity and human health in built environments and the topic requires further research attention.

## Supplementary Information

Supplementary Information.

## Data Availability

Raw sequencing data has been deposited to the Sequence Read Archive (SRA) under BioProject PRJNA757748. The sensitive data that support the findings of this study are available from University of Helsinki but restrictions defined in General Data Protection Regulation (EU 2016/679) and Finnish Data Protection Act 1050/2018 apply to the availability of these data, and so are not publicly available. Data are, however, available from the authors upon reasonable request and with permission from the ethical committee of the local hospital district (Ethical statement number R18026 by Tampereen yliopistollisen sairaalan erityisvastuualue, Pirkanmaa, Finland, the full trial protocol can be requested from the authors).
