# Supplementary material for: Indoor green wall affects health-associated commensal skin microbiota and enhances immune regulation: a randomized trial among urban office workers
Source: Sci Rep. 2022 Apr 20;12:6518. doi: 10.1038/s41598-022-10432-4 (PMC9021224; doi:10.1038/s41598-022-10432-4)
Supplement: Supplementary file 1 — Supplementary Information. [file 41598_2022_10432_MOESM1_ESM.pdf]

SUPPLEMENTARY INFORMATION

Indoor Green Wall affects health-associated commensal skin microbiota and enhances immune regulation – a randomized trial among urban office workers

AUTHOR LIST:

Soininen L[1], Roslund MI[1,3], Nurminen N[2], Puhakka R[1], Laitinen OH[2], Hyöty H[2], Sinkkonen A[3]*, ADELE research group

1Ecosystems and Environment Research Programme, Faculty of Biological and Environmental Sciences, University of Helsinki, Niemenkatu 73, FI-15140 Lahti, Finland.

2Faculty of Medicine and Health Technology, Tampere University, Arvo Ylpön katu 34, FI-33520 Tampere, Finland.

3Natural Resources Institute Finland, Horticulture technologies, Turku and Helsinki, Finland

* Corresponding author

Supporting data for the figures

Supplementary table 1. Supporting data for figures 1, 2 and 4 in this article are given below for the relative abundance of genus Lactobacillus (Lactobacillus), Shannon diversity of Phylum Proteobacteria (Shannon Proteobacteria) and class Gammaproteobacteria (Shannon Gammaproteobacteria) and the concentration of cytokine TGF-β1 (ng/ml). The data include sample size (N), mean, standard deviation (SD), median, minimum value (Min), maximum value (Max) and standard error (SE).

Variable	Time	Treatment	N	Mean	SD	Median	Min	Max	SE
Lactobacillus	t1	Control	17	21.88	27.55	10.00	0.00	87.00	6.68
	t2	Control	17	9.12	12.22	3.00	0.00	40.00	2.96
	t3	Control	16	24.81	61.65	4.50	1.00	248.00	15.41
	t1	Experimental	10	21.90	19.76	12.00	3.00	69.00	6.25
	t2	Experimental	11	31.18	36.02	17.00	1.00	130.00	10.86
	t3	Experimental	10	44.90	86.22	14.50	3.00	286.00	27.27
Shannon Proteobacteria	t1	Control	17	1.54	0.57	1.56	0.56	2.56	0.14
	t2	Control	17	1.58	0.53	1.46	0.84	2.53	0.13
	t3	Control	16	1.50	0.48	1.53	0.64	2.41	0.12
	t1	Experimental	10	1.38	0.55	1.35	0.72	2.71	0.17
	t2	Experimental	11	1.52	0.62	1.52	0.91	2.84	0.19
	t3	Experimental	10	1.71	0.69	1.63	0.70	3.06	0.22
Shannon Gammaproteobacteria	t1	Control	17	1.21	0.50	1.13	0.39	2.00	0.12
	t2	Control	17	1.22	0.45	1.11	0.61	2.21	0.11
	t3	Control	16	1.21	0.37	1.29	0.51	1.89	0.09
	t1	Experimental	10	1.06	0.44	1.02	0.53	2.12	0.14
	t2	Experimental	11	1.24	0.53	1.24	0.68	2.37	0.16
	t3	Experimental	10	1.40	0.54	1.42	0.50	2.31	0.17
TGF-β1	t1	Control	16	24.64	8.54	23.46	9.43	42.57	2.13
	t2	Control	16	19.85	4.53	20.16	9.84	27.17	1.13
	t3	Control	15	22.51	6.71	23.07	10.28	32.66	1.73
	t1	Intervention	10	20.87	5.26	19.39	14.54	32.80	1.66
	t2	Intervention	10	19.45	5.88	17.60	14.94	34.71	1.86
	t3	Intervention	9	24.12	11.31	20.74	16.36	52.65	3.77

Supporting data for the figures

Supplementary table 2. Supporting data for figure 3 in this article are given below for concentration of cytokine IL-17A (pg/ml) and Shannon diversity of Phylum Proteobacteria (Shannon Proteobacteria) and class Gammaproteobacteria (Shannon Gammaproteobacteria. The data are grouped by treatment and city of work (City). The data include sample size (N), mean, standard deviation (SD), median, minimum value (Min), maximum value (Max) and standard error (SE).

Variable	City	Treatment	N	Mean	SD	Median	Min	Max	SE
IL-17A	Lahti	Control	29	3.57	0.97	3.40	2.30	5.90	0.18
	Lahti	Experimental	19	3.31	0.99	3.40	1.70	4.80	0.23
	Tampere	Control	21	3.59	1.22	3.50	2.00	6.50	0.27
	Tampere	Experimental	12	2.85	0.73	2.90	1.60	4.10	0.21
Shannon Proteobacteria	Lahti	Control	29	1.76	0.47	1.73	0.64	2.56	0.09
	Lahti	Experimental	19	1.79	0.63	1.59	0.85	3.06	0.14
	Tampere	Control	21	1.23	0.42	1.14	0.56	2.53	0.09
	Tampere	Experimental	12	1.12	0.29	1.09	0.70	1.60	0.08
Shannon Gammaproteobacteria	Lahti	Control	29	1.39	0.41	1.42	0.51	2.21	0.08
	Lahti	Experimental	19	1.44	0.52	1.29	0.58	2.37	0.12
	Tampere	Control	21	0.97	0.34	0.95	0.39	1.92	0.07
	Tampere	Experimental	12	0.91	0.29	0.90	0.50	1.53	0.08

Shannon diversity of class Gammaproteobacteria and phylum Proteobacteria

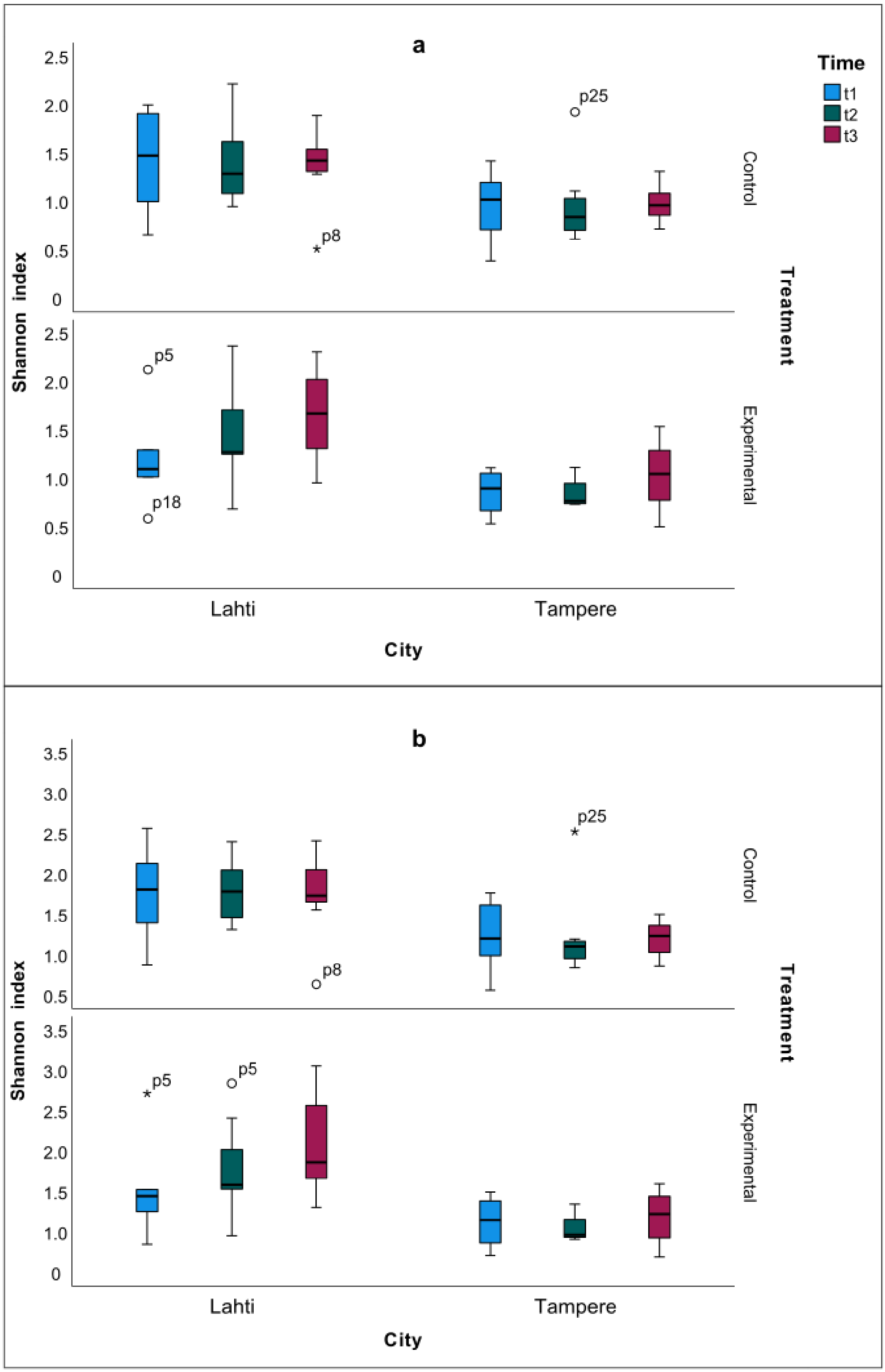

Supplementary fig 1 Shannon diversity index of class Gammaproteobacteria (a) and phylum Proteobacteria (b) divided by cities (x-axis) and treatment (see y-axis). Time: t1= Day0, t2 = Day14, t3 = Day28. Gammaproteobacteria: Lahti P = 0.02, R2 = 0.05, R2random = 0.57, Tampere P = 0.7; Proteobacteria: Lahti P = 0.019, R2 = 0.06, R2random = 0.55, Tampere P = 0.5.

Anti-inflammatory cytokine TGF-β1 concentration and change

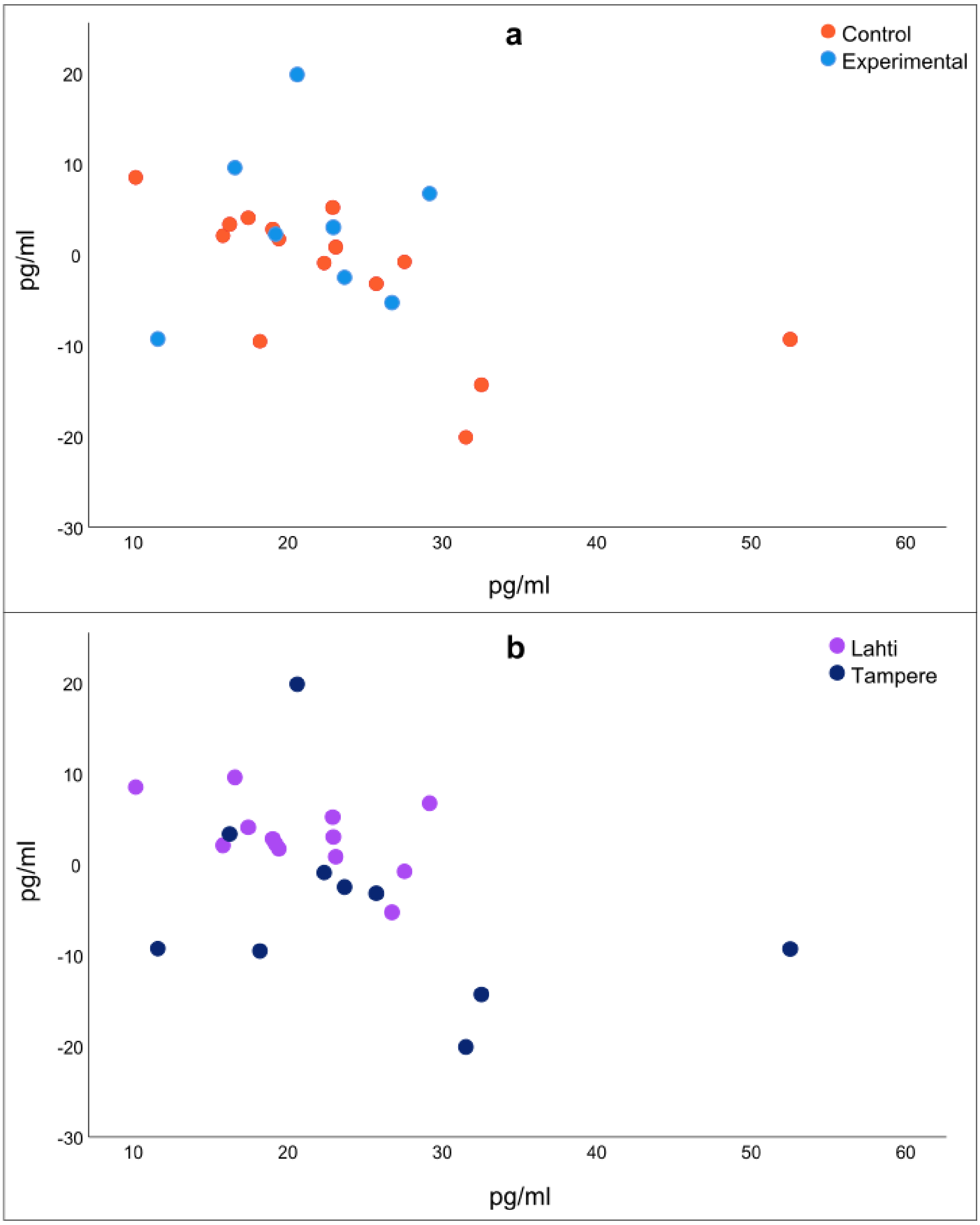

Supplementary fig 2 Anti-inflammatory cytokine TGF-β1: Level of change Day0 – Day28 (y-axis) against concentration (pg/ml) on Day28 by treatment (a) and city (b)
